# Are owners' reports of their dogs’ ‘guilty look’ influenced by the dogs’ action and evidence of the misdeed?

**DOI:** 10.1016/j.beproc.2014.12.010

**Published:** 2015-02

**Authors:** Ljerka Ostojić, Mladenka Tkalčić, Nicola S. Clayton

**Affiliations:** a Department of Psychology, University of Cambridge, Downing Street, Cambridge CB2 3EB, UK; b Department of Psychology, Faculty of Humanities and Social Sciences, University of Rijeka, Sveučilišna avenija 4, 51000 Rijeka Croatia

**Keywords:** Domestic dogs, ‘Guilty look’

## Abstract

While dog owners claim that their dogs’ greeting behaviour after having performed a misdeed indicates the dogs' ‘guilt’, current experimental evidence suggests that dogs show these ‘guilty look’ behaviours as a response to being scolded by their owners. Given reports that ‘guilty look’ behaviours are shown also in the absence of being scolded, we investigated whether the dogs' own actions or the evidence of a misdeed might serve as triggering cues. We manipulated whether or not dogs ate a ‘forbidden’ food item and whether or not the food was visible upon the owners’ return. Based on their dogs’ greeting behaviour, owners stated that their dog had eaten the food no more than expected by chance. In addition, dogs’ greeting behaviours were not affected by their own action or the presence or absence of the food. Thus, our findings do not support the hypothesis that dogs show the ‘guilty look’ in the absence of a concurrent negative reaction by their owners.

## Highlights

• We test cues that trigger dogs’ ‘guilty look’ behaviours.
• The dogs’ action and evidence of the misdeed have no effect on the ‘guilty look’.
• Dogs might not show the ‘guilty look’ in the absence of concurrent scolding by their owners.

## 1 Introduction

Dog owners regularly claim that their dogs exhibit behaviours that indicate that they have performed a misdeed or otherwise disobeyed a rule while the owner was absent (Hecht et al., 2012; Pongrácz et al., 2001). Further, owners state that dogs exhibit these behaviours before owners have discovered the evidence of the misdeed (Hecht et al., 2012). Some owners view these behaviours as an indication that the dogs feel ‘guilty’ (Morris et al., 2008; Hecht et al., 2012; Horowitz, 2009). This view implies that dogs evaluate their own actions according to an internalised code of behaviour or rule (Pongrácz et al., 2001; Sanders, 1993; Morris et al., 2008).

Recently, attempts have been made to disentangle the dogs’ behaviour from anthropomorphic descriptions (Wynne, 2004; 2007) by investigating the context in which dogs perform behaviours associated with this so-called ‘guilty look’. Horowitz (2009) tested dogs in a paradigm in which owners first forbade their dogs to take a food item and then left the test area. The food item was then either eaten by the dogs or removed by the experimenter. The author found that those dogs that were scolded upon their owners' return showed more ‘guilty look’ behaviours than dogs that were greeted by their owners in a friendly manner, regardless of whether the food item had been eaten by the dog or removed by the experimenter. Thus, the author concluded that the dogs' behaviour is best interpreted as the dogs’ fear-response to being scolded (Horowitz, 2009).

An important issue is that if dogs show the ‘guilty look’ out of fear whilst they are being scolded by their owners, then this might happen regardless of what events preceded the scolding event. However, this does not mean that the dogs’ behaviour is never influenced by such preceding events. In line with owners’ claims that dogs show the ‘guilty look’ before the owners found out about the misdeed, the dogs’ behaviours might be triggered by other cues in the total absence of being scolded. Indeed, there is some indication that the dogs’‘guilty look’ behaviours in Horowitz, (2009) study might have also been influenced by whether the food item had been eaten by the dog or had been removed by the experimenter. Those dogs that had not eaten the food but were scolded showed a more intense ‘guilty look’ than dogs that were scolded after they had eaten the food (Horowitz, 2009). It is possible that dogs that had eaten the food expected that they are likely to be scolded whilst dogs that had not eaten the food did not expect to be scolded and were thus more surprised at the scolding event, leading to a more intense fear response (Weiss, 1970).

To test in more detail what triggers the ‘guilty look’ response in the absence of scolding, Hecht et al. (2012) conducted a follow-up study in which owners were asked to assess their dogs’ behaviour and report whether they thought that their dogs had eaten the ‘forbidden’ food item or not. This procedure ensured that individual differences between dogs were taken into account. Overall, owners were able to judge correctly whether their dog had eaten the food or not more than expected by chance. However, two issues make it unlikely that owners’ reports were based predominantly on their dogs’ greeting behaviours; firstly, there was no experimental manipulation of whether dogs had eaten the food or not and secondly, there was a baseline trial in which the owner saw how the dog behaved and in which the owner scolded the dog if it had eaten the food. These two factors make it very likely that owners were basing their judgements on their knowledge of how the dog behaved in the baseline or on any previous occasions that were similar to the test situation (Hecht et al., 2012). The authors attempted to solve this issue by performing an analysis that included only those dogs for which owners were deemed most likely to be basing their judgement on the dogs’ actual greeting behaviour. This analysis revealed that owners could not reliably judge whether their dogs had eaten the ‘forbidden’ food or not.

However, in the absence of a clear experimental manipulation of potential cues, it remains unclear what cues might trigger the ‘guilty look’ in the absence of concurrent scolding. Those cues might be entirely separable from the effect that scolding has on the ‘guilty look’ or they could have previously been associated by dogs with being scolded. In the latter case, dogs might show the ‘guilty look’ when they perceive these predictive cues alone because they expect that they will get scolded by their owners (Lindsay, 2000; Wynne, 2007).

If the ‘guilty look’ was based on some sort of ‘guilt’ as often claimed by dog owners, then the cue triggering this behaviour would have to be linked to the dog’s own action, namely whether the dog has or has not performed the misdeed. However, it has been proposed that another salient cue for the dogs in such situations might be the evidence of the misdeed, regardless of whether the dogs themselves are responsible for it or not (Vollmer, 1977; Wynne, 2007). The aim of the current study was to test whether and which of these two cues might trigger the ‘guilty look’ in the absence of concurrent scolding. Here, we systematically manipulated both the dogs’ action and evidence of the misdeed. The experimenter either removed the food item or let the dogs eat it. In addition, upon the owners’ return, the food item was either absent or it was replaced by the experimenter and thus clearly visible to the owners and, most importantly, to the dogs. Owners were instructed to behave in a neutral manner such that we could investigate whether either or both of these cues might trigger the dogs’ ‘guilty look’ behaviours. Following the procedure used by (Hecht et al. (2012) the dogs’ behaviours were assessed through owners’ statements about whether or not they thought that their dog had eaten the ‘forbidden’ food item. This procedure ensured that individual differences in dogs' greeting behaviours and behaviours that might only be perceivable to the dogs’ owners were taken into account. If the dogs' own action triggers the ‘guilty look’, only owners of those dogs that had eaten the food item should report that their dog had performed the misdeed (regardless of whether the food item was absent or present). By contrast, if evidence of the misdeed triggers the ‘guilty look’, then only those owners for whose dogs the experimenter had not replaced the food item should report that their dog had performed the misdeed (regardless of whether the food item had been eaten by the dog or removed by the experimenter). Finally, if the ‘guilty look’ is triggered by a combination of those cues, then only owners of those dogs that have eaten the food and for whom the food was not replaced by the experimenter should conclude that their dog had performed the misdeed.

## 2 Materials and methods

### 2.1 Subjects

Ninety-six owners and their dogs were tested in Croatia from December 2011 to January 2012 and from June to October 2013 (see Tables in Supplementary Information). Owners were recruited in dog parks, dog schools and training clubs and voluntarily decided to participate in the study. Every owner was tested with one dog only, which ensured independence of the data. Dogs younger than six months and dogs which had spent less than three months with their current owners at the time of testing were not included in the study to ensure a minimum duration during which owners would have had opportunity to learn about their dog’s behaviour in different situations (Horowitz, 2009).

Testing took place in the owner’s home or another indoor or outdoor area that was familiar to the dog to ensure that the dog’s and the owner’s behaviours were not influenced by a novel or uncomfortable context. Every testing room or outdoor area was selected such that it allowed the experimenter and dog to stay in one part while the owner exited the area and had no visual access to the testing area. Thus, only fenced terraces or small gardens were able to serve as adequate outdoor testing areas, in which cases the owner entered the house while the experimenter and dog stayed outside. The experimenter familiarised themselves with the dog prior to testing by playing with it and giving it a treat. Dogs that showed signs of aggression towards humans were not tested (*n* = 1; not included in final sample size). Dogs that were too timid such that they did not want to move freely in the room in the absence of their owner were excluded from the study (*n* = 2; not included in final sample size).

Dogs were not deprived of food prior to the beginning of the experiment. However, if a bowl containing the dog’s food was present in the room, the owners were asked to remove it for the duration of the test. Dogs always had access to water.

Owners were given a box of Burns Pet Nutrition Ltd. Venison Training Treats and a box of Pet Nutrition Venison sausages as appreciation for their participation.

The experiment was approved by the University of Cambridge Animal Ethics Review committee.

### 2.2 Procedure

Prior to testing, the experimenter spent a minimum of ten minutes in the owner’s house. During that time, the experimenter interacted with both the owner and the dog. The dog was further given a piece of the testing food (a piece of ham) to ensure that the dog would in principle eat the food. If the dog did not want to eat the offered food, the test was subsequently conducted using a different treat that was provided by the owner. In these cases, owners were asked to provide two pieces of the test food for the purpose of the experiment.

The experimenter explained the procedure to the owner without revealing the aim of the experiment and gave them the opportunity to ask any questions to clarify the procedure before testing started. The owner was informed that the testing will be recorded and that they could terminate the testing at any point.

After the owner stated that they understood the experimental procedure as explained by the experimenter, they chose a spot in the room where they wanted to place the food item for the dog. The experimenter subsequently pointed the camera towards this area. The owner was given one piece of the test food which they placed on the chosen spot and forbid the dog to take it. The owner was instructed to forbid the dog to take the food in any way they wanted. If the dog took the food item before the owner left the area (*n* = 6), the owners were immediately instructed to repeat the procedure until the dog would not touch the food while the owner was still in the room. Thus, owners did not scold their dog for having taken the food, although this might have affected the way they issued the command to not take the food on the next trial. Once the owner left the room, the experimenter either removed the food item immediately (*Not Eaten & Not Replaced* and *Not Eaten & Replaced* groups) or did not do anything such that the dog could take the food (*Eaten & Not Replaced* and *Eaten & Replaced* groups). Dogs were randomly assigned to one of these four testing groups. In cases where the dog was tested in the *Eaten* groups but did not immediately eat the food, the experimenter attempted to increase the salience of the food item by touching it or tapping the area next to it. The experimenter never handed the food to the dog. After the owner was called back in, they were reminded to not speak with the dog or touch it but to simply observe it for 10s. Subsequently, the experimenter asked the owner whether they thought that the dog had eaten the food item or whether they thought that the experimenter had removed it. After their reply, the owner was debriefed about the purpose of the experiment.

### 2.3 Analysis

The data were collected by three experimenters (two female: LO, YK and one male: MF). Each experimenter tested one third of owners and dogs in each of the four testing groups to ensure that potential differences between the groups could not be accounted by the different numbers of individuals tested by the different experimenters (see Tables in Supplementary Information). We recorded the owner’s response regarding whether they thought that their dog had eaten the food or that the food had been removed by the experimenter.

The owner's response in the task was analysed using binomial tests and general linearized mixed models (GLMMs) with a binary logit structure using Genstat version 13.1 (VSN International, Hemel Hempstead, UK). Wald χ^2^ statistics (Bolker et al., 2009) and *p* values were obtained from models containing all explanatory terms. The dispersion parameter could not be fixed at 1 and was thus estimated as reported in Table 1 (Zuur et al., 2009). All tests were two-directional (two-tailed *p* values). Alpha was set at 0.05.

## 3 Results

In each of the four groups, owners stated that their dog’s behaviour indicated that the dog has disobeyed the rule no more than expected by chance (binomial test, *n* = 24, *Eaten & Replaced:* 15/24, *p* = 0.3075; *Eaten & Not Replaced*: 11/24, *p* = 0.8388; *Not Eaten & Replaced*: 11/24, *p* = 0.8388; *Not Eaten & Not Replaced*: 15/24, *p* = 0.3075, individual data see Tables in Supplementary Information).

**Fig. 1:**
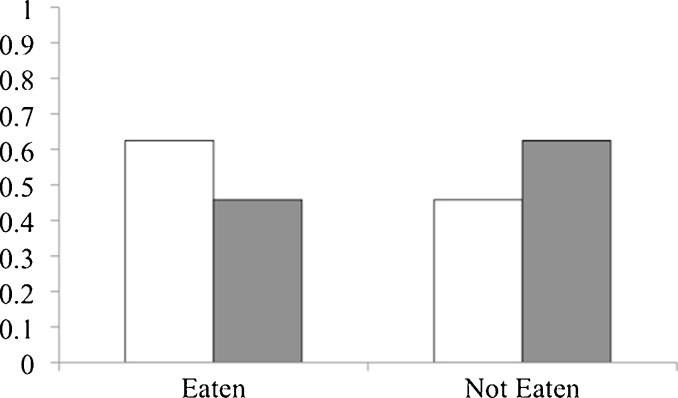
Proportion of dogs for whom owners reported that they had eaten the ‘forbidden’ food item. The left two columns depict the owners whose dogs ate the food item (Eaten) and the right two columns depict the owners of dogs for whom the experimenter removed the food item (Not Eaten). White bars depict the owners for whose dogs the food item was present upon their return (Replaced) and grey bars depict owners for whose dogs the food item was absent upon their return (Not Replaced). Chance level is at 0.05.

To test whether the owners’ responses differed between the groups, we ran a GLMM with *action* (Eaten, Not Eaten) and *presence of food* (Replaced, Not Replaced) as fixed factors and experimenter identity as a random factor (Table 1). The owners’ responses were not affected by whether or not the dog had eaten the food (*action*: χ^2^ = 0.00, *p* > 0.999) nor by whether or not the food item was replaced by the experimenter (*presence of food*: χ^2^ = 0.00, *p* > 0.999). In addition, the owners’ responses were similar regardless of whether the food item had been replaced after the dog had eaten it or after it had been taken away by the experimenter (*action* x *presence of food*: χ^2^ = 2.60, *p* = 0.111, Fig. 1).

## 4 Discussion

Based on their dogs’ greeting behaviours, owners did not judge that their dogs had eaten the ‘forbidden’ food item more often than expected by chance. Critically, the dogs’ behaviour as perceived by the owners did not differ between the different conditions, suggesting that the dogs’ ‘guilty look’ was not influenced by their own action (i.e. whether the food had been eaten by the dog or removed by the experimenter) or the evidence of the misdeed (i.e. whether the food was present or absent upon the owners’ return).

Our findings indicate that–in the absence of scolding–neither of the two cues that were experimentally manipulated trigger the ‘guilty look’ in dogs. Importantly, these two cues have been proposed as the two most salient cues for dogs in such situations. Our results further rule out the possibility that the whole context in which dogs were tested (owners issuing a command, leaving the area and then returning) might have served as triggering cues, as owners in all experimental groups reported that the dogs had eaten the food not more than expected by chance. Thus, our findings could be taken to support the hypothesis that dogs’ ‘guilty look’ behaviours depend on their owners’ concurrent behaviour such as scolding or other negative reactions (Horowitz, 2009). Horowitz (2009) found some indication that dogs’ ‘guilty look’ behaviours might be affected by whether they themselves had eaten a ‘forbidden’ food item or whether it had been removed by the experimenter because dogs showed a more intense ‘guilty look’ response to being scolded in the latter case. Critically, this effect was only found in combination with the dogs being scolded by their owners at the same time. It thus seems necessary for future studies to first validate owners’ claims that dogs show ‘guilty look’ behaviours before the owners are aware of any misdeed and thus before owners can react in any negative way.

Even though we emphasise the importance of an experimental manipulation of the different cues that might trigger the ‘guilty look’, it is possible that both in the current and in previous experiments with such manipulations, the dogs’ behaviours were affected by the presence of the experimenter. Recent studies have shown that if dogs are given a particular command or have learned a particular rule, they are more likely to forget or ignore the command or rule after an interfering event, for example when the owner leaves the area and another person enters (Topál et al., 2009) or even when the owner themselves leave and subsequently return (Hertel et al., 2014). Such retroactive interference effects on individuals’ memory are well documented in both humans and other animals (Fuster and Bauer, 1974; Grant, 1988; Marcovitch and Zelazo, 2003; Yoon et al., 2006). In the case of owners issuing a command, leaving and then returning, the experimenter’s presence and their manipulations might cause interference with the dogs’ memory for the owners' command and thus the dogs' behaviour upon the owners’ return. Although it is likely that any event that happens after the owners’ command will lead to retroactive interference, it is possible that a social agent might cause a higher level of interference. In addition, in those rare occasions in which the dogs did not immediately eat the food and the experimenter attempted to increase the salience of the food by tapping at the area around it, dogs might have interpreted this situation as the experimenter ‘allowing’ them to eat the food, which might have–for the dogs–rendered the owner’s command ineffective. Thus, to eliminate the effects of interference caused by the experimenter, future studies could manipulate the dogs’ action and the evidence of the misdeed using an automated procedure.

## 5 Conclusion

The dogs’ behaviour as perceived by their owners was not affected by the dogs’ own action or evidence of the misdeed. Thus, our findings could be taken to suggest that these two cues do not trigger the ‘guilty look’ in dogs, at least not in situations in which they are not paired with concurrent scolding by the owners. However, due to the possibility of retrospective interference effects caused by the presence of an experimenter, a study in which the experimental manipulations are conducted using an automated procedure might be necessary to provide a definite answer regarding whether the ‘guilty look’ is shown outside of a scolding event and if so, which cues trigger this specific behaviour in dogs.

## Figures and Tables

**Table 1:** GLMM analysis of the factors affecting the owners’ reports regarding whether they thought that their dogs had eaten the food.

	Wald statistic	d*f*	*P*
Full model			
Action (Eaten, Not Eaten)	0.00	1	>.999
Presence of food (Replaced, Not Replaced)	0.00	1	>0.999
Action * Presence of food	2.60	1	0.111

Data were fitted using a binomial distribution with a logit-link function. Experimenter identity was fitted as a random term (estimated variance component for subject in the minimal model = 0.168, SE = 0.243). Estimated dispersion parameter: 0.154.
