# Supplementary material for: Are owners' reports of their dogs’ ‘guilty look’ influenced by the dogs’ action and evidence of the misdeed?
Source: Behav Processes. 2015 Feb;111:97–100. doi: 10.1016/j.beproc.2014.12.010 (PMC4310318; doi:10.1016/j.beproc.2014.12.010)
Supplement: Supplementary file 1 [file mmc1.docx]

| Dogs tested in the ***Eaten & Not Replaced*** group | | | | | |
| --- | --- | --- | --- | --- | --- |
| Dog’s name | Dog’s breed | Dog’s gender | Dog’s age | Owner’s gender | Owner’s response |
| Brie | Australian Shepherd | F | 3 | F | No |
| Erika | Mixed breed | F | 1 | F | No |
| Don | Labrador Retriever | M | 9 | F | Yes |
| Ruby | Mixed breed | F | 11 | F | Yes |
| Ben | Mixed breed | M | 11 | F | Yes |
| Nika | Mixed breed | F | 8 | F | No |
| Abigail | Golden Retriever | F | 8 | F | Yes |
| Tonko | Mixed breed | M | 6 | M | Yes |
| Rem | Labrador Retriever | M | 11 | F | No |
| Koni | Pekinese | F | 2 | F | No |
| Nike | Mixed breed | M | 10 | F | Yes |
| Leo | Golden Retriever | M | 9 | M | Yes |
| Thor | Doberman | M | 1 | M | No |
| Nala | Cane Corso | F | 1 | F | No |
| Nera | Mixed breed | F | 7 | M | Yes |
| Oz | Basset | M | 7 | F | No |
| Pako | Middle Schnauzer | M | 14 | F | Yes |
| Pepi | Maltese | F | 5 | F | No |
| Benjo | Golden Retriever | M | 6 | F | Yes |
| Onyx | King Charles Spaniel | M | 4 | M | No |
| Nala | Labrador Retriever | F | 10 | F | No |
| Bart | Mixed breed | M | 7 | F | No |
| Molly | English Bulldog | F | 2 | F | No |
| Kozmo | Mixed breed | M | 7 | M | yes |
| The column ‘Dog’s age’ indicates the dog’s age in years. F = female, M = male. The column ‘Owner’s response’ indicates the owners’ answer to the question whether thy think that their dog has eaten the food item. The light gray rows represent dogs tested by experimenter LO, the white rows represent dogs tested by experimenter YK and the dark grey ones represent dogs tested by experimenter MF. | | | | | |

| Dogs tested in the ***Eaten & Replaced*** group | | | | | |
| --- | --- | --- | --- | --- | --- |
| Dog’s name | Dog’s breed | Dog’s gender | Dog’s age | Owner’s gender | Owner’s response |
| Bak | American Cocker Spaniel | M | * | F | No |
| Hara | Golden Retriever | F | 8 | F | Yes |
| Nordi | Samojede | M | 2 | M | No |
| Abigail | Ridgeback | F | 1 | M | Yes |
| Kea | Stafford Terrier | F | 1 | M | Yes |
| Malicka | Mixed breed | F | 7 | F | Yes |
| Kan | Boxer | M | 1 | M | Yes |
| Kozmo | Middle Snauzer | M | 5 | F | No |
| Sara | Mixed breed | F | 4 | M | Yes |
| Miška | Mixed breed | F | 8 | F | Yes |
| Pas | Huskie | F | * | M | Yes |
| Whiskey | Mixed breed | M | 5 | M | No |
| Vanda | Poodle | F | 5 | F | Yes |
| Ugo | Malteser | M | 7 | F | No |
| Chiara | Mixed breed | F | 8 | F | Yes |
| Ginger | Cane Corso | F | 2 | F | No |
| Areta | Springer Spaniel | F | 11 | M | Yes |
| Ron | Welsh terrier | M | 12 | M | Yes |
| Chilli | French Bulldog | M | 3 | M | Yes |
| Viki | Border Collie | M | 4 | M | No |
| Gita | Mixed breed | F | 2 | M | No |
| Ayra | Hovawart | F | 1 | F | No |
| Luksa | Malinois | F | 7 | F | Yes |
| Khal | Labrador Retriever | M | 9 | F | Yes |
| The column ‘Dog’s age’ indicates the dog’s age in years. F = female, M = male. * denotes dogs whose age is unknown. The column ‘Owner’s response’ indicates the owners’ answer to the question whether thy think that their dog has eaten the food item. The light gray rows represent dogs tested by experimenter LO, the white rows represent dogs tested by experimenter YK and the dark grey ones represent dogs tested by experimenter MF. | | | | | |

| Dogs tested in the ***Not*** ***Eaten & Not Replaced*** group | | | | | |
| --- | --- | --- | --- | --- | --- |
| Dog’s name | Dog’s breed | Dog’s gender | Dog’s age | Owner’s gender | Owner’s response |
| Zara | Mixed breed | F | 6 | F | Yes |
| Apri | Poodle | F | 3 | F | Yes |
| Rita | French Bulldog | F | 2? | F | Yes |
| Tina | Mixed breed | F | 5 | F | Yes |
| Balu | Pug | M | 2? | F | No |
| Bela | Mixed breed | F | 9 | F | No |
| Dona | German Shepherd | F | 2 | F | No |
| Tricket | Poodle | M | 1 | M | Yes |
| Leda | Labrador Retriever | F | 8 | M | Yes |
| Mili1 | Mixed breed | F | 3 | M | Yes |
| Pablo | Golden Retriever | M | 11 | F | Yes |
| LaVie | Shih Tzu | M | 2 | F | Yes |
| Cor | mixed breed | M | 2 | F | Yes |
| Bobi | mixed breed | M | 9 | F | Yes |
| Darwin | Labrador Retriever | M | 2 | F | No |
| Ria | Cane Corso | F | 3 | F | Yes |
| Klara | Schnauzer | F | 1 | F | No |
| Artur | Mixed breed | M | 2 | M | No |
| Kiara | Golden Retriever | F | 7 | F | Yes |
| Otto | Mixed breed | M | 7 | F | No |
| Roxie | Labrador Retriever | F | 2 | M | No |
| Moro | Mixed breed | M | 14 | F | No |
| Lu | Boxer | F | 1 | F | Yes |
| Kimbo | Mixed breed | M | 11 | M | Yes |
| The column ‘Dog’s age’ indicates the dog’s age in years. F = female, M = male. The column ‘Owner’s response’ indicates the owners’ answer to the question whether thy think that their dog has eaten the food item. The light gray rows represent dogs tested by experimenter LO, the white rows represent dogs tested by experimenter YK and the dark grey ones represent dogs tested by experimenter MF. | | | | | |

| Dogs tested in the ***Not Eaten & Replaced*** group | | | | | |
| --- | --- | --- | --- | --- | --- |
| Dog’s name | Dog’s breed | Dog’s gender | Dog’s age | Owner’s gender | Owner’s response |
| Simba | West Highland White Terrier | M | 9 | F | No |
| Rama | Labrador Retriever | F | 7 | F | No |
| Ozzy | Mixed breed | M | 1 | F | Yes |
| Cupko | Mixed breed | M | 1 | M | Yes |
| Monk | Bobtail | M | 2 | F | No |
| Marley | Mixed breed | M | 3 | F | Yes |
| Mousey | Mixed breed | F | 3 | F | Yes |
| Nera | Mixed breed | F | 8 | F | No |
| Mili 2 | Chihuaha | F | 1 | F | Yes |
| Gabi | Poodle | F | 2 | F | Yes |
| Gigi | Pekinese | F | 4 | M | Yes |
| Sani | Mixed breed | F | 4 | M | No |
| Kiara | King Charles Spaniel | F | 4 | F | Yes |
| Delboy | Bulldog | M | 3 | F | Yes |
| Neo | Mixed breed | M | 2 | M | No |
| Frida | Shih Tzu | F | 4 | F | No |
| Bubi | Border Collie | M | 2 | M | No |
| Nora | Labrador Retriever | F | 8 | F | No |
| Tonkica | Mixed breed | F | 1 | F | Yes |
| Ogi | West Highland White Terrier | M | 7 | M | No |
| Spillo | Yorkshire Terrier | M | 6 | F | No |
| Rina | Pekinese | F | 5 | F | No |
| Don | German Shepherd | M | 2 | M | Yes |
| Mozart | Mixed breed | M | 12 | F | No |
| The column ‘Dog’s age’ indicates the dog’s age in years. F = female, M = male. The column ‘Owner’s response’ indicates the owners’ answer to the question whether thy think that their dog has eaten the food item. The light gray rows represent dogs tested by experimenter LO, the white rows represent dogs tested by experimenter YK and the dark grey ones represent dogs tested by experimenter MF. | | | | | |
